# Prevalence and incidence of bronchiectasis in Italy

**DOI:** 10.1186/s12890-020-1050-0

**Published:** 2020-01-16

**Authors:** Stefano Aliberti, Giovanni Sotgiu, Francesco Lapi, Andrea Gramegna, Claudio Cricelli, Francesco Blasi

**Affiliations:** 1 Fondazione IRCCS Ca’ Granda Ospedale Maggiore Policlinico, Respiratory Unit and Cystic Fibrosis Adult Center, Milan, Italy; 2 Department of Pathophysiology and Transplantation, University of Milan, Milan, Italy; 3 Clinical Epidemiology and Medical Statistics Unit, Department of Clinical and Experimental Medicine, University of Sassari, Sassari, Italy; 4 Health Search, Italian College of General Practitioners and Primary Care, Florence, Italy; 5 Italian College of General Practitioners and Primary Care, Florence, Italy

**Keywords:** Bronchiectasis, Epidemiology, Italy

## Abstract

**Background:**

The understanding of the epidemiology of bronchiectasis is still affected by major limitations with very few data published worldwide. The aim of this study was to estimate the epidemiological burden of bronchiectasis in Italy in the adult population followed-up by primary care physicians.

**Methods:**

This study analyzed data coming from a large primary care database with 1,054,376 subjects in the period of time 2002–2015. Patients with bronchiectasis were selected by the use of International Statistical Classification of Diseases, 9th revision, Clinical Modification codes (ICD-9-CM).

**Results:**

Patients with bronchiectasis were more likely to have a history of tuberculosis (0.47% vs. 0.06%, *p* < 0.0001), had higher rates of asthma (16.6% vs. 6.2%, *p* < 0.0001), COPD (23.3% vs. 6.4%, p < 0.0001) and rheumatoid arthritis (1.9% vs. 0.8%, p < 0.0001). The prevalence and incidence of bronchiectasis in primary care in Italy in 2015 were 163 per 100,000 population and 16.3 per 100,000 person-years, respectively. Prevalence and incidence increased with age and overall rates were highest in men over 75 years old. Prevalence and incidence computed after the exclusion of patients with a diagnosis of either asthma or COPD is 130 per 100,000 and 11.1 cases per 100,000 person-years, respectively.

**Conclusions:**

Bronchiectasis is not a rare condition in Italian adult population. Further studies are needed to confirm our results and provide a better insight on etiology of bronchiectasis in Italy.

**Trial registration:**

not applicable.

## Background

Bronchiectasis is a chronic respiratory syndrome following a permanent dilation of the bronchi, associated with the occurrence of cough, daily sputum production, and recurrent respiratory infections [1]. The clinical syndrome is the final outcome of several genetic and acquired medical conditions [2]. It has a substantial healthcare and societal impact, mainly generated by frequent hospitalizations and mortality [3, 4].

A large clinical and epidemiological heterogeneity can be found across continents and among countries within the same continent. Currently, the epidemiological burden of the disease is partially unknown with only cross-sectional and retrospective studies been published until now (based in the USA and a few European countries) [5–9]. Furthermore, data sources are medical insurance or hospital discharge databases, or partially representative ad hoc population-based studies [3, 4, 6, 7].

Old-fashioned epidemiological reports highlighted bronchiectasis as a rare disease. On the contrary, a recent UK longitudinal study, based on primary care data, showed an increase in annual incidence since 2004 [5]. It is reasonable to assume that bronchiectasis epidemiology might be variable and local data would be needed to define national policy priorities and to guide research and development activities for new drugs. So far, no epidemiological studies have been conducted in Italy in inpatient and outpatient settings.

In addition, the co-existence of bronchiectasis with other obstructive pulmonary diseases, such as asthma and chronic obstructive pulmonary diseases (COPD), might hinder the precise assessment of bronchiectasis epidemiology; by now no studies have evaluated prevalence and incidence in a population of patients with only bronchiectasis and no concomitant diagnosis of other chronic respiratory diseases.

The aim of this study was to estimate the epidemiological burden of bronchiectasis in Italy in the adult (aged > 14 years) population followed-up by primary care physicians, stratified by age and gender, both in overall population and excluding patients with a concomitant diagnosis of either asthma or COPD.

## Methods

### Data source

Data were retrieved from computer-based records included in the Health Search IMS Health Longitudinal Database (HSD). The HSD, which complies with the European Union guidelines on the use of medical data for research purposes, collected clinical records from 1996 to 2015. Patients recruited in the system had an anonymous code linked to demographic information, medical records, and date of death. Diseases were classified according to the International Classification of Disease, 9th revision, Clinical Modification (ICD-9-CM). HSD population is comparable with the Italian population surveyed by the Italian National Institute of Statistics (ISTAT) for the following variables: gender, age, and geographical location.

### Study population

The study population included patients aged > 14 years and followed-up up to 31 December 2015 by 800 Italian general practitioners (GPs). They were detected with the ICD9CM codes 494 and 011.5. The recruitment period was from 1st January 2002 to 31st December 2015. Patients with diagnosis of ‘suspected bronchiectasis’ AND/OR patients with diagnosis of cystic fibrosis were excluded. Since this study was based on open-access anonymized data, approval from ethical committees was not needed. Each case was matched up to ten controls, who were randomly selected in the whole cohort, according to sex, age, year of cohort entry and duration of follow-up.

### Data collection

Primary outcomes included prevalence and incidence of bronchiectasis in the Italian adult population. Clinical characteristics were reported for all included patients. Information on conditions associated with bronchiectasis were also retrieved, including COPD (491.2; 493.2), asthma (493.9), primary ciliary dyskinesia (759.3), tuberculosis (011.9), Kartagener syndrome (759.3), HIV infection (V08, 042), rheumatoid arthritis (714.0), inflammatory bowel disease as ulcerative colitis (556.9) and Crohn disease (555.9), bone marrow transplant (41.0), hypogammaglobinemia (279.0), allergic bronchopulmonary aspergillosis ABPA (518.6), common variable immunodeficiency (279.06), and alpha1-antitrypsin deficiency (273.4).

### Study definitions

The prevalence of bronchiectasis was estimated by calculating the proportion of patients with a bronchiectasis diagnosis from 1st January 2002 to 31st December 2015 to the adult population (per 100,000) with the 95% confidence interval (95% CI). The annual incidence rate from 2002 to 2015 was estimated by dividing new cases of bronchiectasis to person-years with 95% CI according to annual registration. A new case of bronchiectasis was a patient with a new ICD diagnosis of bronchiectasis without any similar diagnoses the years before. Both prevalence and incidence estimates were stratified by both gender and age classes each single year of analysis. Sensitivity analysis was conducted to evaluate incidence and prevalence estimates in patients with a diagnosis of neither COPD nor asthma.

## Results

### Demographics and clinical characteristics of adult patients with bronchiectasis

Mean (SD) age of patients with bronchiectasis was 67.5 (14.4) years, without any statistically significant differences in comparison with the control group (Table 1). Patients with bronchiectasis were more likely to have a history of tuberculosis (0.47% VS. 0.06%; *P* < 0.0001), had higher prevalence of asthma (16.6% VS. 6.2%; *P* < 0.0001), COPD (23.3% VS. 6.4%; *P* < 0.0001), and rheumatoid arthritis (1.9% VS. 0.8%; *P* < 0.0001), as summarized in Table 1.

**Table 1:** Conditions associated with bronchiectasis in our cohort in 2015

Associated conditions	Cases	Controls	*p*
History of tuberculosis	8 (0.47)	10 (0.06)	< 0.0001
Asthma	285 (16.60)	1068 (6.22)	< 0.0001
COPD	400 (23.26)	1099 (6.40)	< 0.0001
HIV	0 (0)	17 (0.10)	ns
Rheumatoid arthritis	33 (1.92)	135 (0.79)	< 0.0001
Ulcerative colitis	9 (0.52)	69 (0.40)	ns
Crohn disease	0 (0)	5 (0.03)	ns
Hypogammaglobinemia	1 (0.06)	5 (0.03)	ns
Common variable immunodeficiency	1 (0.06)	0 (0)	ns

### Prevalence and incidence of bronchiectasis in the Italian adult population

**Fig. 1:**
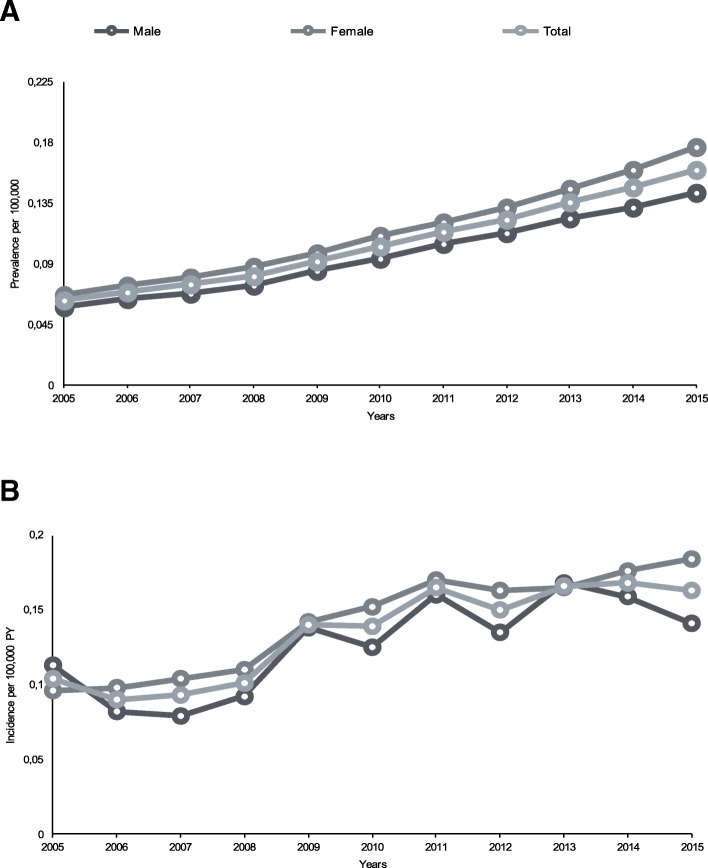
**A** Trend in annual prevalence of bronchiectasis by sex from 2005 to 2015. **B** Trend in annual incidence of bronchiectasis by sex from 2005 to 2015

A total of 1,054,376 subjects (543,974, 52%, females and 268,693, 25%, aged > 65 years) were included in the database. Temporal trends of bronchiectasis incidence and prevalence by gender are summarized in Fig. 1.

**Fig. 2:**
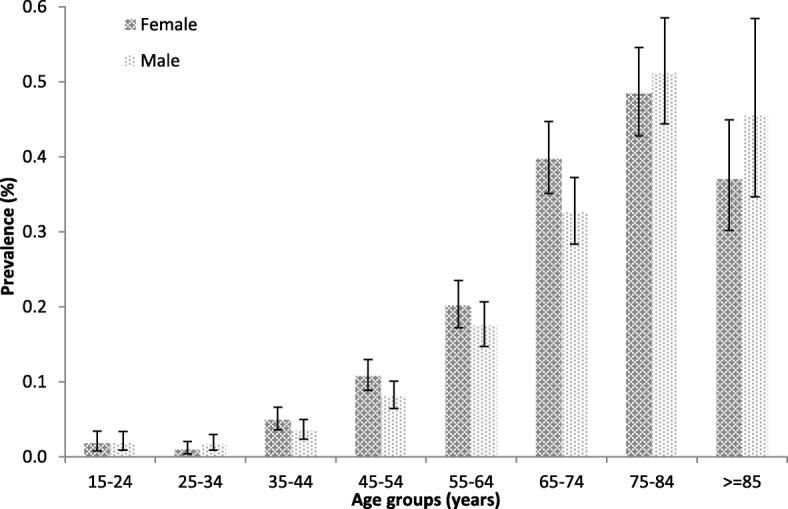
Mean prevalence of bronchiectasis by age group and sex in Italy in 2015

Prevalence increased in the overall study population, rising from 62 in 2005 to 163 per 100,000 population in 2015. In the last year of the analysis, it was higher in women than in men (178 VS. 147 per 100,000 population, respectively). Prevalence also increased with age in the overall population and in both males and females to the highest rate of 466 cases per 100,000 population (497 and 446 per 100,000 population in men and women, respectively) in patients aged > 75 years. The incidence of bronchiectasis in 2015 was 16.3 cases per 100,000 person-years, with a higher rate among women (18.2 VS. 14.1 per 100,000 person-years). Age was related to increased incidence, with a maximum rate of 42.9 per 100,000 person-years in patients aged 75–84 years (46.7 and 40.0 per 100,000 person-years in men and women, respectively). Prevalence and incidence of bronchiectasis by age group and gender is described in Fig. 2.

### Prevalence and incidence of bronchiectasis in adults excluding patients with a diagnosis of either asthma or COPD

In 2015, the prevalence was 130 cases per 100,000 population, resulting higher in women than in men (140 VS. 110 per 100,000 population, respectively). Increasing age was linked to increased prevalence both in the overall population and in males and females up to the highest rate of 350 cases per 100,000 population in patients > 75 years (340 and 352 per 100,000 population in men and women, respectively).

**Fig. 3:**
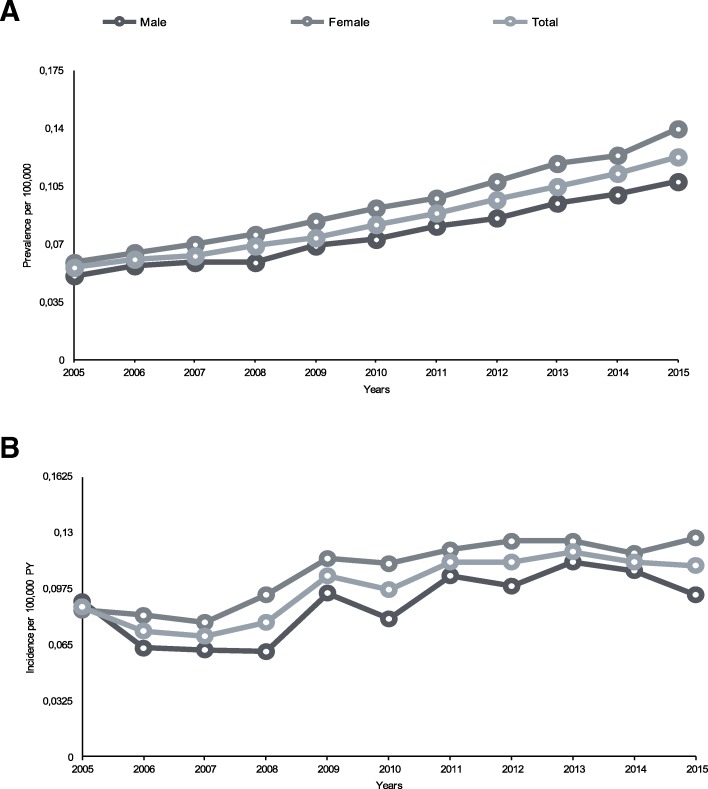
**A** Trend in annual prevalence of bronchiectasis by sex from 2005 to 2015, excluding those with a diagnosis of either asthma or COPD. **B** Trend in annual incidence of bronchiectasis by sex from 2005 to 2015, excluding those with a diagnosis of either asthma or COPD

**Fig. 4:**
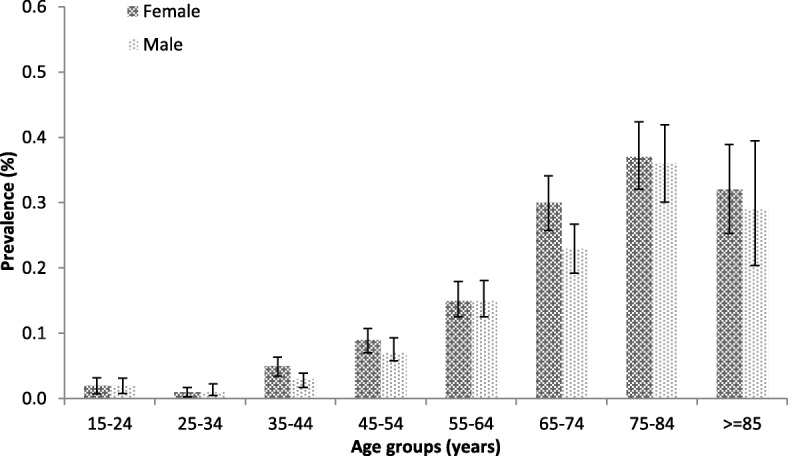
Mean prevalence of bronchiectasis by age group and sex in Italy in 2015, excluding those with a diagnosis of either asthma or COPD

The incidence of bronchiectasis in 2015 was 11.1 cases per 100,000 person-years, with higher rate in women than men (12.7 VS. 9.4 per 100,000 person-years, respectively). Incidence increased with aging up to a maximum rate of 28.9 per 100,000 person-years in patients aged 75–84 years (29.0 in men and 28.8 in women). Data are summarized in Figs. 3 and 4.

## Discussion

Bronchiectasis overall prevalence in the Italian population referring to GPs is 163 per 100,000 population, whereas annual incidence is 16.3 per 100,000 person-years. Both prevalence and incidence increase with age, with the highest rates reported in patients aged > 75 years. Prevalence and incidence computed after the exclusion of patients with a diagnosis of either asthma or COPD is 130 per 100,000 and 11.1 person-years, respectively.

This study provides the first epidemiological report on bronchiectasis in Italy, showing that it is not a rare disease. Italian estimates seem similar to other European settings, ranked between the lower prevalence described by Ringshausen et al. in Germany (67 cases per 100,000 population) and the higher UK prevalence (566 and 485 new cases per 100,000 population in women and men, respectively) [5, 7]. Interestingly, Italian prevalence is lower if compared with the only other bronchiectasis cohort recruited in Southern Europe. In 2012 Monteagudo analyzed primary care medical reports of 5.8 million people in Catalonia and reported on a prevalence of 362 patients per 100,000 population [8]. This inconsistency might be related to several reasons, including different data sources and algorithms for data collection; however, geographical heterogeneity might have a key role, as recently highlighted by Chandrasekaran [10]. A better understanding of the epidemiological variability should be achieved by the analysis of international multicentric cohorts, recruited with the same methodology and using the same clinical definitions.

While Italian bronchiectasis prevalence and incidence are slightly higher in females across all age groups, different estimates were found in 2015 in people older than 75 years (prevalence 511 VS. 484 per 100,000 population and incidence 46.7 VS. 40.0 per 100,000 person-years in males and females, respectively). The increased rates in males are consistent with findings from similar studies and - as already observed - might be attributed to the high proportion of COPD patients in this age class [5, 8]. The analysis in patients without a concomitant diagnosis of COPD confirmed this hypothesis, where it was found that females were more prevalent in all age groups. Notably, in our dataset COPD is the most prevalent (23.3%) condition related to bronchiectasis. At present, though biological plausibility and epidemiological association has been reported, the definition of COPD as a cause of bronchiectasis is not widely accepted [11, 12]. A recent literature review showed conflicting bronchiectasis prevalence estimates in COPD populations, ranging from 4 to 28%, partially explained by the enrollment of different populations or different CT diagnostic criteria [13]. While the interpretation of a simple co-existence or co-morbidity between COPD and bronchiectasis still holds a prominent position, it has been recently postulated that these medical conditions might occur as an overlap syndrome (the acronym BCOS has been proposed) with possible consequences in terms of treatment and increased mortality [14–16].

Annual prevalence of bronchiectasis increased from 2005 to 2015 in men and women, as well as in the overall population and in population without asthma and COPD, in agreement with previous findings [5, 6]. While some of bronchiectasis could still be post-infective, this increase in incidence among elderly people might be attributed to specific adult-onset etiologies, that are becoming more and more prevalent in aged and chronically-ill patients. However, the increasing trend might be partly explained by the wider use of chest CT scan, population ageing, as well as increased awareness of bronchiectasis among respiratory physicians.

This study has both strengths and limitations. It provides robust data on epidemiology of bronchiectasis in Italy; they are collected from GPs and provide a real-life and population-based overview. However, the use of ICD codes and the retrospective study design are likely to underestimate the real prevalence and incidence of the disease. In addition, ICD codes analysis is not related to a reliable data collection on the underlying etiology. Finally, although radiology is needed for bronchiectasis diagnosis, we could not prove if each diagnosis was supported by chest CT scan. As a consequence, diagnosis accuracy might be limited.

In addition, the study design did not allow to investigate risk factors for bronchiectasis development, but only medical conditions associated with bronchiectasis have been reported.

## Conclusions

Bronchiectasis is not a rare condition in Italy. Our results are the first epidemiological report of bronchiectasis prevalence in Italy and contribute to a better assessment of bronchiectasis epidemiology in Southern Europe. Additional analysis from national registries are needed to confirm study findings and could provide insights on bronchiectasis etiology in Italy.

## Data Availability

The raw data of this paper cannot be shared, according to the policy of Health Search IMS Health Longitudinal Database (HSD).

## Abbreviations

COPD: Chronic obstructive pulmonary diseases
HSD: Health Search IMS Health Longitudinal Database
